# A new coordination tetra­mer of copper(I) iodide and benzyl­dimethyl­amine: tetra-μ_3_-iodido-tetra­kis[(benzyl­dimethyl­amine-κ*N*)copper(I)]

**DOI:** 10.1107/S1600536809026208

**Published:** 2009-07-11

**Authors:** Shuying Yang, Yuebao Li, Yujie Cui, Jianguo Pan

**Affiliations:** a State Key Laboratory Base of Novel Functional Materials and Preparation Science, Faculty of Materials Science and Chemical Engineering, Ningbo University, Ningbo, Zhejiang 315211, People’s Republic of China

## Abstract

The title compound, [Cu_4_I_4_(C_9_H_13_N)_4_], has a distorted cubane-like [Cu_4_I_4_] core structure. Each Cu^I^ atom is tetra­hedrally coordinated by three I atoms and one N atom of an benzyl­dimethyl­amine ligand. Each I atom acts as a μ_3_-ligand, linking three Cu^I^ atoms. The Cu—I bond distances vary between 2.6328 (7) and 2.7121 (6) Å, while the Cu—N bond distances vary between 2.107 (3) and 2.122 (3) Å.

## Related literature

For the synthesis and structures of copper iodide coordination polymers, see: Bi *et al.* (2007*a* ▶,*b* ▶); Chen *et al.* (2008 ▶).
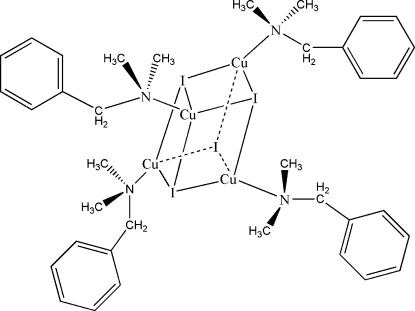


## Experimental

### 

#### Crystal data

[Cu_4_I_4_(C_9_H_13_N)_4_]
*M* *_r_* = 1302.58
Monoclinic, 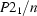

*a* = 17.758 (4) Å
*b* = 11.544 (2) Å
*c* = 21.540 (4) Å
β = 100.16 (3)°
*V* = 4346.3 (15) Å^3^
*Z* = 4
Mo *K*α radiation
μ = 4.80 mm^−1^
*T* = 298 K
0.38 × 0.29 × 0.27 mm

#### Data collection

Rigaku R-AXIS RAPID diffractometer
Absorption correction: multi-scan (*ABSCOR*; Higashi, 1995 ▶) *T*_min_ = 0.719, *T*_max_ = 1.000 (expected range = 0.197–0.274)
40151 measured reflections
9884 independent reflections
8944 reflections with *I* > 2σ(*I*)
*R*_int_ = 0.063

#### Refinement

*R*[*F* ^2^ > 2σ(*F* ^2^)] = 0.031
*wR*(*F* ^2^) = 0.059
*S* = 1.08
9884 reflections
434 parameters
6 restraints
H-atom parameters constrained
Δρ_max_ = 1.77 e Å^−3^
Δρ_min_ = −0.94 e Å^−3^

### 

Data collection: *RAPID-AUTO* (Rigaku, 1998 ▶); cell refinement: *RAPID-AUTO*; data reduction: *CrystalStructure* (Rigaku/MSC, 2002 ▶); program(s) used to solve structure: *SHELXS97* (Sheldrick, 2008 ▶); program(s) used to refine structure: *SHELXL97* (Sheldrick, 2008 ▶); molecular graphics: *ORTEPII* (Johnson, 1976 ▶); software used to prepare material for publication: *SHELXL97*.

## Supplementary Material

Crystal structure: contains datablocks I, global. DOI: 10.1107/S1600536809026208/fj2223sup1.cif

Structure factors: contains datablocks I. DOI: 10.1107/S1600536809026208/fj2223Isup2.hkl

Additional supplementary materials:  crystallographic information; 3D view; checkCIF report

## Figures and Tables

**Table 1:** Selected geometric parameters (Å, °)

Cu1—N1	2.107 (3)
Cu1—I4	2.6711 (8)
Cu1—I1	2.6892 (6)
Cu1—I2	2.6953 (8)
Cu2—N2	2.108 (3)
Cu2—I3	2.6609 (6)
Cu2—I4	2.6750 (6)
Cu2—I1	2.6819 (9)
Cu3—N3	2.122 (3)
Cu3—I4	2.6611 (6)
Cu3—I2	2.6947 (8)
Cu3—I3	2.7121 (6)
Cu4—N4	2.112 (3)
Cu4—I1	2.6328 (7)
Cu4—I2	2.6788 (6)
Cu4—I3	2.7090 (10)

**Fig. 1.**
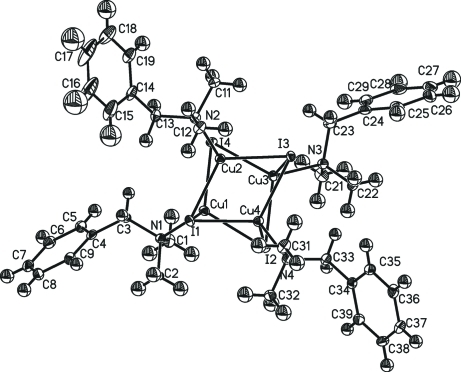
The symmetric unit structure of Cu4I4(C9H13N)4 with displacement ellipsoids drawn at the 45% probability level.

**Fig. 2.**
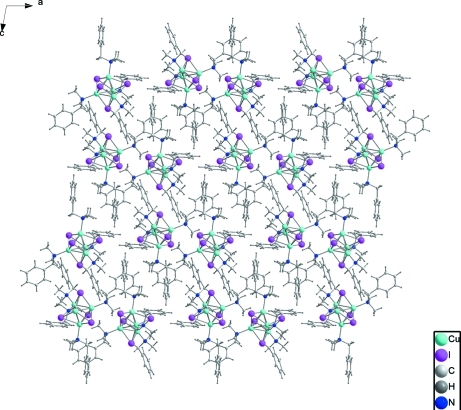
The supramolecular structure formed via intermolecular forces.

## supplementary crystallographic information

### Comment

Copper halides have been widely investigated due to their rich photoluminescent properties and intriguing topology. In recent years, great efforts have been taken to synthesize and characterize copper iodide coordination polymers which have special crystal structure and properties. By now several classes of copper iodide coordination polymers have been successfully synthesized and investigated (Bi *et al.*, 2007*a*,*b*; Chen *et al.*, 2008). Herein, we report the synthesis and crystal structure of the title complex.

The title compound (Fig.1) has a distorted cubanelike [Cu_4_I_4_] core structure. Each copper(I) atom is tetrahedrally coordinated by three iodide atoms and one N atom of n-benzyldimethylamine. Each iodide atom acting as µ_3_-I links three copper(I) atoms. The Cu—I bond distances vary between 2.6328 (7) and 2.7121 (6) Å, while the Cu—N bond distances vary between 2.107 (3) and 2.122 (3) Å. The Cu—I and Cu—N bond distances correspond well with that found in the literatures listed in the comment. Finally, Cu_4_I_4_(C_9_H_13_N)_4_ forms the supramolecular structure *via* intermolecular forces.

### Experimental

Cuprous iodide (0.3881 g) and n-benzyldimethylamine (1 ml) were sealed in a glass vial, which was heated at 353 K for two days. Then, the solution (0.5 ml) was put in another glass vial. One day later colorless and transparent crystals were obtained by slow evaporation of the solution at 353 K.

### Refinement

All H atoms associated with C atoms were positioned geometrically and refined as riding model [C—H1=0.93 Å, C—H2=0.97 Å, C—H3=0.96Å *U*_iso_(H)=1.2*U*_eq_(C), *U*_iso_(H)=1.5*U*_eq_(C)].

### Crystal data

**Table:**

[Cu_4_I_4_(C_9_H_13_N)_4_]	*F*(000) = 2496
*M**_r_* = 1302.58	*D*_x_ = 1.991 Mg m^−^^3^
Monoclinic, *P*2_1_/*n*	Mo *K*α radiation, λ = 0.71073 Å
Hall symbol: -P 2yn	Cell parameters from 40151 reflections
*a* = 17.758 (4) Å	θ = 3.0–27.4°
*b* = 11.544 (2) Å	µ = 4.80 mm^−^^1^
*c* = 21.540 (4) Å	*T* = 298 K
β = 100.16 (3)°	Block, colorless
*V* = 4346.3 (15) Å^3^	0.38 × 0.29 × 0.27 mm
*Z* = 4	

### Data collection

**Table:**

Rigaku R-AXIS RAPID diffractometer	9884 independent reflections
Radiation source: fine-focus sealed tube	8944 reflections with *I* > 2σ(*I*)
graphite	*R*_int_ = 0.063
ω scans	θ_max_ = 27.4°, θ_min_ = 3.0°
Absorption correction: multi-scan (*ABSCOR*; Higashi, 1995)	*h* = −23→23
*T*_min_ = 0.719, *T*_max_ = 1.000	*k* = −14→14
40151 measured reflections	*l* = −27→27

### Refinement

**Table:**

Refinement on *F*^2^	Secondary atom site location: difference Fourier map
Least-squares matrix: full	Hydrogen site location: inferred from neighbouring sites
*R*[*F*^2^ > 2σ(*F*^2^)] = 0.031	H-atom parameters constrained
*wR*(*F*^2^) = 0.059	*w* = 1/[σ^2^(*F*_o_^2^) + 6.5262*P*] where *P* = (*F*_o_^2^ + 2*F*_c_^2^)/3
*S* = 1.08	(Δ/σ)_max_ = 0.003
9884 reflections	Δρ_max_ = 1.77 e Å^−^^3^
434 parameters	Δρ_min_ = −0.94 e Å^−^^3^
6 restraints	Extinction correction: *SHELXL97* (Sheldrick, 2008), Fc^*^=kFc[1+0.001xFc^2^λ^3^/sin(2θ)]^-1/4^
Primary atom site location: structure-invariant direct methods	Extinction coefficient: 0.00015 (4)

### Special details

**Table:**

Geometry. All e.s.d.'s (except the e.s.d. in the dihedral angle between two l.s. planes) are estimated using the full covariance matrix. The cell e.s.d.'s are taken into account individually in the estimation of e.s.d.'s in distances, angles and torsion angles; correlations between e.s.d.'s in cell parameters are only used when they are defined by crystal symmetry. An approximate (isotropic) treatment of cell e.s.d.'s is used for estimating e.s.d.'s involving l.s. planes.
Refinement. Refinement of *F*^2^ against ALL reflections. The weighted *R*-factor *wR* and goodness of fit *S* are based on *F*^2^, conventional *R*-factors *R* are based on *F*, with *F* set to zero for negative *F*^2^. The threshold expression of *F*^2^ > σ(*F*^2^) is used only for calculating *R*-factors(gt) *etc*. and is not relevant to the choice of reflections for refinement. *R*-factors based on *F*^2^ are statistically about twice as large as those based on *F*, and *R*- factors based on ALL data will be even larger.

### Fractional atomic coordinates and isotropic or equivalent isotropic displacement parameters (Å^2^)

**Table:**

	*x*	*y*	*z*	*U*_iso_*/*U*_eq_	
Cu1	−0.00846 (2)	0.35843 (3)	0.16475 (2)	0.01613 (9)	
Cu2	0.12653 (2)	0.45686 (3)	0.18503 (2)	0.01504 (9)	
Cu3	0.05779 (2)	0.42114 (3)	0.289283 (19)	0.01594 (9)	
Cu4	0.00552 (2)	0.58059 (3)	0.20498 (2)	0.01611 (9)	
I1	0.020897 (11)	0.530945 (17)	0.088571 (10)	0.01611 (6)	
I2	−0.093901 (11)	0.439045 (17)	0.247110 (10)	0.01551 (6)	
I3	0.144530 (11)	0.610927 (17)	0.278368 (10)	0.01425 (5)	
I4	0.110408 (11)	0.244188 (17)	0.230246 (10)	0.01544 (6)	
C1	−0.1133 (2)	0.1608 (3)	0.14134 (18)	0.0230 (7)	
H1A	−0.1468	0.1099	0.1140	0.034*	
H1B	−0.1415	0.1990	0.1695	0.034*	
H1C	−0.0724	0.1166	0.1653	0.034*	
C2	−0.14480 (18)	0.3176 (3)	0.06819 (17)	0.0218 (7)	
H2A	−0.1784	0.2680	0.0401	0.033*	
H2B	−0.1248	0.3767	0.0443	0.033*	
H2C	−0.1727	0.3529	0.0975	0.033*	
C3	−0.03673 (17)	0.1916 (3)	0.05871 (16)	0.0170 (7)	
H3A	0.0047	0.1479	0.0834	0.020*	
H3B	−0.0140	0.2515	0.0364	0.020*	
C4	−0.08217 (17)	0.1108 (3)	0.01039 (16)	0.0172 (7)	
C5	−0.0909 (2)	−0.0059 (3)	0.02337 (18)	0.0212 (7)	
H5A	−0.0688	−0.0356	0.0625	0.025*	
C6	−0.13257 (19)	−0.0788 (3)	−0.02167 (18)	0.0237 (8)	
H6A	−0.1385	−0.1565	−0.0122	0.028*	
C7	−0.16496 (19)	−0.0367 (3)	−0.08022 (18)	0.0239 (8)	
H7A	−0.1924	−0.0859	−0.1102	0.029*	
C8	−0.15646 (19)	0.0791 (3)	−0.09414 (18)	0.0232 (8)	
H8A	−0.1783	0.1081	−0.1335	0.028*	
C9	−0.11522 (18)	0.1522 (3)	−0.04909 (17)	0.0200 (7)	
H9A	−0.1096	0.2298	−0.0588	0.024*	
C11	0.29409 (18)	0.4446 (3)	0.20081 (17)	0.0192 (7)	
H11A	0.3412	0.4481	0.1849	0.029*	
H11B	0.2889	0.3695	0.2187	0.029*	
H11C	0.2943	0.5028	0.2327	0.029*	
C12	0.2371 (2)	0.5829 (3)	0.12264 (17)	0.0207 (7)	
H12A	0.2836	0.5870	0.1058	0.031*	
H12B	0.2385	0.6392	0.1556	0.031*	
H12C	0.1943	0.5985	0.0897	0.031*	
C13	0.2257 (2)	0.3751 (3)	0.09826 (17)	0.0221 (7)	
H13A	0.1764	0.3808	0.0707	0.027*	
H13B	0.2283	0.2996	0.1182	0.027*	
C14	0.2872 (2)	0.3810 (3)	0.05807 (18)	0.0266 (8)	
C15	0.2717 (3)	0.4335 (4)	−0.0012 (2)	0.0582 (16)	
H15A	0.2229	0.4620	−0.0160	0.070*	
C16	0.3270 (4)	0.4439 (5)	−0.0382 (3)	0.0761 (19)	
H16A	0.3156	0.4807	−0.0771	0.091*	
C17	0.3996 (4)	0.4000 (4)	−0.0177 (3)	0.0686 (18)	
H17A	0.4370	0.4071	−0.0428	0.082*	
C18	0.4163 (3)	0.3456 (4)	0.0402 (2)	0.0439 (11)	
H18A	0.4649	0.3157	0.0543	0.053*	
C19	0.3596 (2)	0.3359 (3)	0.07735 (19)	0.0281 (8)	
H19A	0.3709	0.2981	0.1160	0.034*	
C21	0.0141 (2)	0.3227 (3)	0.40695 (18)	0.0243 (8)	
H21A	0.0179	0.3227	0.4520	0.036*	
H21B	0.0306	0.2491	0.3935	0.036*	
H21C	−0.0381	0.3361	0.3874	0.036*	
C22	0.03421 (19)	0.5276 (3)	0.40854 (17)	0.0207 (7)	
H22A	0.0360	0.5268	0.4533	0.031*	
H22B	−0.0176	0.5391	0.3875	0.031*	
H22C	0.0657	0.5893	0.3977	0.031*	
C23	0.14486 (18)	0.3971 (3)	0.41885 (17)	0.0217 (7)	
H23A	0.1753	0.4590	0.4054	0.026*	
H23B	0.1625	0.3249	0.4034	0.026*	
C24	0.15991 (19)	0.3937 (3)	0.49034 (18)	0.0249 (8)	
C25	0.17937 (19)	0.4938 (4)	0.52562 (19)	0.0316 (9)	
H25A	0.1844	0.5634	0.5050	0.038*	
C26	0.1913 (2)	0.4913 (4)	0.5908 (2)	0.0378 (10)	
H26A	0.2045	0.5587	0.6137	0.045*	
C27	0.1834 (2)	0.3883 (5)	0.6217 (2)	0.0398 (11)	
H27A	0.1906	0.3869	0.6656	0.048*	
C28	0.1650 (2)	0.2878 (4)	0.58806 (19)	0.0362 (10)	
H28A	0.1604	0.2185	0.6091	0.043*	
C29	0.1533 (2)	0.2902 (4)	0.52248 (18)	0.0295 (9)	
H29A	0.1409	0.2222	0.4998	0.035*	
C31	0.0059 (2)	0.8294 (3)	0.17982 (18)	0.0238 (8)	
H31A	−0.0159	0.9056	0.1787	0.036*	
H31B	0.0122	0.8073	0.1381	0.036*	
H31C	0.0549	0.8294	0.2072	0.036*	
C32	−0.1203 (2)	0.7451 (3)	0.16079 (18)	0.0246 (8)	
H32A	−0.1433	0.8204	0.1604	0.037*	
H32B	−0.1531	0.6888	0.1752	0.037*	
H32C	−0.1130	0.7255	0.1189	0.037*	
C33	−0.05426 (19)	0.7741 (3)	0.26976 (16)	0.0186 (7)	
H33A	−0.0045	0.7671	0.2966	0.022*	
H33B	−0.0875	0.7164	0.2835	0.022*	
C34	−0.08608 (19)	0.8926 (3)	0.28009 (16)	0.0171 (7)	
C35	−0.03778 (19)	0.9878 (3)	0.29292 (18)	0.0224 (7)	
H35A	0.0147	0.9775	0.2960	0.027*	
C36	−0.0660 (2)	1.0976 (3)	0.30126 (18)	0.0233 (8)	
H36A	−0.0328	1.1603	0.3088	0.028*	
C37	−0.1438 (2)	1.1136 (3)	0.29828 (17)	0.0220 (7)	
H37A	−0.1629	1.1869	0.3045	0.026*	
C38	−0.19297 (19)	1.0205 (3)	0.28603 (17)	0.0214 (7)	
H38A	−0.2453	1.0312	0.2839	0.026*	
C39	−0.16446 (19)	0.9105 (3)	0.27686 (17)	0.0189 (7)	
H39A	−0.1980	0.8484	0.2685	0.023*	
N1	−0.08119 (14)	0.2486 (2)	0.10298 (13)	0.0157 (6)	
N2	0.22903 (15)	0.4653 (2)	0.14853 (13)	0.0158 (6)	
N3	0.06292 (15)	0.4150 (2)	0.38841 (13)	0.0162 (6)	
N4	−0.04546 (15)	0.7461 (2)	0.20371 (13)	0.0171 (6)	

### Atomic displacement parameters (Å^2^)

**Table:**

	*U*^11^	*U*^22^	*U*^33^	*U*^12^	*U*^13^	*U*^23^
Cu1	0.01373 (18)	0.01576 (18)	0.0179 (2)	−0.00103 (16)	0.00007 (15)	−0.00207 (15)
Cu2	0.01317 (18)	0.01511 (18)	0.0169 (2)	−0.00067 (15)	0.00288 (15)	−0.00087 (15)
Cu3	0.01647 (19)	0.01768 (19)	0.0138 (2)	0.00067 (16)	0.00297 (15)	0.00108 (15)
Cu4	0.01831 (19)	0.01386 (18)	0.0161 (2)	0.00297 (16)	0.00278 (15)	0.00088 (15)
I1	0.01885 (10)	0.01741 (10)	0.01135 (11)	−0.00066 (8)	0.00072 (8)	0.00055 (8)
I2	0.01233 (10)	0.01651 (10)	0.01800 (12)	0.00070 (8)	0.00350 (8)	−0.00110 (8)
I3	0.01502 (10)	0.01455 (10)	0.01326 (11)	−0.00241 (8)	0.00275 (7)	−0.00213 (7)
I4	0.01374 (10)	0.01215 (9)	0.02016 (12)	0.00196 (8)	0.00229 (8)	0.00118 (8)
C1	0.0227 (17)	0.0228 (17)	0.025 (2)	−0.0068 (15)	0.0082 (14)	−0.0037 (14)
C2	0.0144 (15)	0.0229 (17)	0.026 (2)	0.0043 (14)	−0.0024 (13)	−0.0055 (14)
C3	0.0113 (14)	0.0206 (15)	0.0190 (18)	−0.0016 (13)	0.0025 (12)	−0.0043 (13)
C4	0.0114 (14)	0.0207 (16)	0.0210 (19)	−0.0002 (13)	0.0066 (12)	−0.0047 (13)
C5	0.0228 (17)	0.0171 (15)	0.023 (2)	0.0033 (14)	0.0018 (14)	−0.0027 (13)
C6	0.0213 (17)	0.0179 (16)	0.032 (2)	−0.0015 (14)	0.0062 (15)	−0.0055 (14)
C7	0.0179 (16)	0.0252 (17)	0.029 (2)	−0.0020 (15)	0.0046 (14)	−0.0115 (15)
C8	0.0189 (16)	0.0320 (19)	0.0186 (19)	−0.0016 (15)	0.0032 (14)	−0.0050 (15)
C9	0.0207 (16)	0.0197 (16)	0.0208 (19)	−0.0018 (14)	0.0070 (13)	−0.0022 (13)
C11	0.0144 (15)	0.0217 (16)	0.0216 (19)	0.0030 (13)	0.0039 (13)	−0.0006 (13)
C12	0.0246 (17)	0.0164 (15)	0.023 (2)	0.0022 (14)	0.0102 (14)	0.0037 (13)
C13	0.0245 (17)	0.0197 (16)	0.022 (2)	0.0025 (15)	0.0030 (14)	−0.0068 (14)
C14	0.039 (2)	0.0215 (17)	0.022 (2)	0.0121 (17)	0.0131 (16)	0.0004 (14)
C15	0.094 (4)	0.059 (3)	0.025 (3)	0.051 (3)	0.021 (3)	0.011 (2)
C16	0.139 (5)	0.061 (3)	0.044 (3)	0.061 (3)	0.059 (3)	0.030 (3)
C17	0.121 (5)	0.044 (3)	0.062 (4)	0.026 (3)	0.076 (3)	0.019 (2)
C18	0.051 (3)	0.038 (2)	0.052 (3)	0.010 (2)	0.034 (2)	0.004 (2)
C19	0.037 (2)	0.0242 (18)	0.026 (2)	0.0077 (17)	0.0160 (16)	0.0042 (15)
C21	0.0254 (18)	0.0253 (18)	0.022 (2)	−0.0069 (16)	0.0042 (14)	0.0059 (15)
C22	0.0193 (16)	0.0251 (17)	0.0189 (19)	0.0020 (15)	0.0069 (13)	−0.0016 (14)
C23	0.0148 (15)	0.0314 (18)	0.0195 (19)	0.0052 (15)	0.0042 (13)	0.0052 (15)
C24	0.0130 (15)	0.042 (2)	0.020 (2)	0.0067 (16)	0.0032 (13)	0.0017 (16)
C25	0.0154 (16)	0.052 (2)	0.027 (2)	−0.0037 (18)	0.0019 (15)	−0.0029 (18)
C26	0.0194 (18)	0.067 (3)	0.026 (2)	0.000 (2)	0.0035 (16)	−0.012 (2)
C27	0.0194 (18)	0.082 (3)	0.019 (2)	0.013 (2)	0.0049 (15)	0.002 (2)
C28	0.0246 (19)	0.063 (3)	0.022 (2)	0.016 (2)	0.0071 (15)	0.015 (2)
C29	0.0240 (18)	0.044 (2)	0.021 (2)	0.0129 (17)	0.0057 (15)	0.0046 (17)
C31	0.0315 (19)	0.0167 (15)	0.025 (2)	0.0036 (15)	0.0112 (15)	0.0066 (14)
C32	0.0249 (18)	0.0231 (17)	0.024 (2)	0.0081 (15)	−0.0021 (14)	−0.0026 (14)
C33	0.0231 (16)	0.0173 (15)	0.0162 (18)	0.0047 (14)	0.0062 (13)	0.0018 (13)
C34	0.0205 (16)	0.0165 (15)	0.0146 (17)	0.0015 (14)	0.0038 (13)	0.0000 (12)
C35	0.0154 (15)	0.0268 (18)	0.025 (2)	−0.0008 (15)	0.0033 (14)	−0.0070 (15)
C36	0.0241 (17)	0.0201 (16)	0.026 (2)	−0.0077 (15)	0.0069 (15)	−0.0082 (14)
C37	0.0260 (18)	0.0179 (16)	0.024 (2)	0.0031 (15)	0.0088 (14)	−0.0017 (14)
C38	0.0147 (15)	0.0264 (17)	0.024 (2)	0.0032 (14)	0.0044 (13)	0.0003 (14)
C39	0.0169 (15)	0.0197 (16)	0.0204 (19)	−0.0029 (14)	0.0044 (13)	−0.0009 (13)
N1	0.0117 (12)	0.0168 (13)	0.0187 (15)	−0.0010 (11)	0.0026 (10)	−0.0023 (11)
N2	0.0169 (13)	0.0131 (12)	0.0179 (15)	−0.0009 (11)	0.0043 (11)	0.0002 (11)
N3	0.0158 (13)	0.0183 (13)	0.0149 (15)	0.0004 (11)	0.0034 (11)	0.0029 (11)
N4	0.0205 (14)	0.0167 (13)	0.0143 (15)	0.0042 (12)	0.0039 (11)	0.0023 (11)

### Geometric parameters (Å, °)

**Table:**

Cu1—N1	2.107 (3)	C14—C19	1.381 (5)
Cu1—Cu2	2.6189 (7)	C14—C15	1.396 (6)
Cu1—I4	2.6711 (8)	C15—C16	1.374 (8)
Cu1—I1	2.6892 (6)	C15—H15A	0.9300
Cu1—I2	2.6953 (8)	C16—C17	1.383 (9)
Cu1—Cu4	2.7043 (7)	C16—H16A	0.9300
Cu1—Cu3	2.8266 (9)	C17—C18	1.381 (7)
Cu2—N2	2.108 (3)	C17—H17A	0.9300
Cu2—I3	2.6609 (6)	C18—C19	1.397 (6)
Cu2—I4	2.6750 (6)	C18—H18A	0.9300
Cu2—Cu4	2.6774 (7)	C19—H19A	0.9300
Cu2—I1	2.6819 (9)	C21—N3	1.473 (4)
Cu2—Cu3	2.7694 (9)	C21—H21A	0.9600
Cu3—N3	2.122 (3)	C21—H21B	0.9600
Cu3—Cu4	2.6368 (7)	C21—H21C	0.9600
Cu3—I4	2.6611 (6)	C22—N3	1.488 (4)
Cu3—I2	2.6947 (8)	C22—H22A	0.9600
Cu3—I3	2.7121 (6)	C22—H22B	0.9600
Cu4—N4	2.112 (3)	C22—H22C	0.9600
Cu4—I1	2.6328 (7)	C23—N3	1.501 (4)
Cu4—I2	2.6788 (6)	C23—C24	1.516 (5)
Cu4—I3	2.7090 (10)	C23—H23A	0.9700
C1—N1	1.484 (4)	C23—H23B	0.9700
C1—H1A	0.9600	C24—C25	1.393 (6)
C1—H1B	0.9600	C24—C29	1.396 (5)
C1—H1C	0.9600	C25—C26	1.382 (6)
C2—N1	1.474 (4)	C25—H25A	0.9300
C2—H2A	0.9600	C26—C27	1.382 (7)
C2—H2B	0.9600	C26—H26A	0.9300
C2—H2C	0.9600	C27—C28	1.377 (7)
C3—N1	1.494 (4)	C27—H27A	0.9300
C3—C4	1.519 (4)	C28—C29	1.391 (5)
C3—H3A	0.9700	C28—H28A	0.9300
C3—H3B	0.9700	C29—H29A	0.9300
C4—C5	1.391 (5)	C31—N4	1.480 (4)
C4—C9	1.396 (5)	C31—H31A	0.9600
C5—C6	1.394 (5)	C31—H31B	0.9600
C5—H5A	0.9300	C31—H31C	0.9600
C6—C7	1.379 (5)	C32—N4	1.479 (4)
C6—H6A	0.9300	C32—H32A	0.9600
C7—C8	1.385 (5)	C32—H32B	0.9600
C7—H7A	0.9300	C32—H32C	0.9600
C8—C9	1.392 (5)	C33—N4	1.494 (4)
C8—H8A	0.9300	C33—C34	1.510 (4)
C9—H9A	0.9300	C33—H33A	0.9700
C11—N2	1.484 (4)	C33—H33B	0.9700
C11—H11A	0.9600	C34—C35	1.392 (5)
C11—H11B	0.9600	C34—C39	1.397 (5)
C11—H11C	0.9600	C35—C36	1.386 (5)
C12—N2	1.483 (4)	C35—H35A	0.9300
C12—H12A	0.9600	C36—C37	1.384 (5)
C12—H12B	0.9600	C36—H36A	0.9300
C12—H12C	0.9600	C37—C38	1.380 (5)
C13—N2	1.496 (4)	C37—H37A	0.9300
C13—C14	1.510 (5)	C38—C39	1.393 (5)
C13—H13A	0.9700	C38—H38A	0.9300
C13—H13B	0.9700	C39—H39A	0.9300
			
N1—Cu1—Cu2	143.85 (8)	N2—C11—H11A	109.5
N1—Cu1—I4	112.06 (8)	N2—C11—H11B	109.5
Cu2—Cu1—I4	60.74 (2)	H11A—C11—H11B	109.5
N1—Cu1—I1	102.92 (8)	N2—C11—H11C	109.5
Cu2—Cu1—I1	60.68 (2)	H11A—C11—H11C	109.5
I4—Cu1—I1	117.89 (2)	H11B—C11—H11C	109.5
N1—Cu1—I2	105.65 (8)	N2—C12—H12A	109.5
Cu2—Cu1—I2	110.21 (2)	N2—C12—H12B	109.5
I4—Cu1—I2	107.88 (2)	H12A—C12—H12B	109.5
I1—Cu1—I2	109.76 (2)	N2—C12—H12C	109.5
N1—Cu1—Cu4	141.67 (7)	H12A—C12—H12C	109.5
Cu2—Cu1—Cu4	60.368 (17)	H12B—C12—H12C	109.5
I4—Cu1—Cu4	106.24 (2)	N2—C13—C14	116.4 (3)
I1—Cu1—Cu4	58.436 (18)	N2—C13—H13A	108.2
I2—Cu1—Cu4	59.485 (14)	C14—C13—H13A	108.2
N1—Cu1—Cu3	149.25 (8)	N2—C13—H13B	108.2
Cu2—Cu1—Cu3	60.99 (3)	C14—C13—H13B	108.2
I4—Cu1—Cu3	57.82 (2)	H13A—C13—H13B	107.3
I1—Cu1—Cu3	107.241 (19)	C19—C14—C15	117.5 (4)
I2—Cu1—Cu3	58.36 (2)	C19—C14—C13	122.7 (3)
Cu4—Cu1—Cu3	56.893 (14)	C15—C14—C13	119.9 (4)
N2—Cu2—Cu1	141.53 (8)	C16—C15—C14	121.5 (5)
N2—Cu2—I3	104.72 (8)	C16—C15—H15A	119.3
Cu1—Cu2—I3	113.60 (3)	C14—C15—H15A	119.3
N2—Cu2—I4	109.47 (7)	C15—C16—C17	120.3 (5)
Cu1—Cu2—I4	60.592 (18)	C15—C16—H16A	119.8
I3—Cu2—I4	110.16 (2)	C17—C16—H16A	119.8
N2—Cu2—Cu4	143.63 (7)	C18—C17—C16	119.5 (5)
Cu1—Cu2—Cu4	61.40 (2)	C18—C17—H17A	120.2
I3—Cu2—Cu4	60.99 (2)	C16—C17—H17A	120.2
I4—Cu2—Cu4	106.90 (2)	C17—C18—C19	119.5 (5)
N2—Cu2—I1	103.19 (8)	C17—C18—H18A	120.2
Cu1—Cu2—I1	60.96 (2)	C19—C18—H18A	120.2
I3—Cu2—I1	110.28 (2)	C14—C19—C18	121.6 (4)
I4—Cu2—I1	118.005 (16)	C14—C19—H19A	119.2
Cu4—Cu2—I1	58.845 (19)	C18—C19—H19A	119.2
N2—Cu2—Cu3	147.38 (8)	N3—C21—H21A	109.5
Cu1—Cu2—Cu3	63.21 (3)	N3—C21—H21B	109.5
I3—Cu2—Cu3	59.886 (17)	H21A—C21—H21B	109.5
I4—Cu2—Cu3	58.490 (13)	N3—C21—H21C	109.5
Cu4—Cu2—Cu3	57.88 (2)	H21A—C21—H21C	109.5
I1—Cu2—Cu3	109.12 (2)	H21B—C21—H21C	109.5
N3—Cu3—Cu4	131.57 (8)	N3—C22—H22A	109.5
N3—Cu3—I4	119.92 (7)	N3—C22—H22B	109.5
Cu4—Cu3—I4	108.51 (2)	H22A—C22—H22B	109.5
N3—Cu3—I2	101.81 (8)	N3—C22—H22C	109.5
Cu4—Cu3—I2	60.31 (2)	H22A—C22—H22C	109.5
I4—Cu3—I2	108.19 (2)	H22B—C22—H22C	109.5
N3—Cu3—I3	100.94 (8)	N3—C23—C24	115.5 (3)
Cu4—Cu3—I3	60.84 (2)	N3—C23—H23A	108.4
I4—Cu3—I3	109.03 (2)	C24—C23—H23A	108.4
I2—Cu3—I3	117.263 (17)	N3—C23—H23B	108.4
N3—Cu3—Cu2	150.92 (7)	C24—C23—H23B	108.4
Cu4—Cu3—Cu2	59.313 (18)	H23A—C23—H23B	107.5
I4—Cu3—Cu2	58.983 (17)	C25—C24—C29	118.3 (4)
I2—Cu3—Cu2	105.82 (3)	C25—C24—C23	121.0 (4)
I3—Cu3—Cu2	58.072 (16)	C29—C24—C23	120.7 (4)
N3—Cu3—Cu1	152.01 (7)	C26—C25—C24	121.0 (4)
Cu4—Cu3—Cu1	59.22 (2)	C26—C25—H25A	119.5
I4—Cu3—Cu1	58.159 (17)	C24—C25—H25A	119.5
I2—Cu3—Cu1	58.38 (3)	C25—C26—C27	119.9 (4)
I3—Cu3—Cu1	105.80 (2)	C25—C26—H26A	120.1
Cu2—Cu3—Cu1	55.798 (19)	C27—C26—H26A	120.1
N4—Cu4—I1	107.50 (8)	C28—C27—C26	120.4 (4)
N4—Cu4—Cu3	137.59 (8)	C28—C27—H27A	119.8
I1—Cu4—Cu3	114.89 (2)	C26—C27—H27A	119.8
N4—Cu4—Cu2	146.59 (8)	C27—C28—C29	119.8 (4)
I1—Cu4—Cu2	60.66 (3)	C27—C28—H28A	120.1
Cu3—Cu4—Cu2	62.81 (2)	C29—C28—H28A	120.1
N4—Cu4—I2	104.45 (8)	C28—C29—C24	120.7 (4)
I1—Cu4—I2	112.03 (2)	C28—C29—H29A	119.7
Cu3—Cu4—I2	60.92 (2)	C24—C29—H29A	119.7
Cu2—Cu4—I2	108.94 (2)	N4—C31—H31A	109.5
N4—Cu4—Cu1	147.00 (8)	N4—C31—H31B	109.5
I1—Cu4—Cu1	60.494 (14)	H31A—C31—H31B	109.5
Cu3—Cu4—Cu1	63.89 (2)	N4—C31—H31C	109.5
Cu2—Cu4—Cu1	58.236 (17)	H31A—C31—H31C	109.5
I2—Cu4—Cu1	60.089 (18)	H31B—C31—H31C	109.5
N4—Cu4—I3	103.60 (7)	N4—C32—H32A	109.5
I1—Cu4—I3	110.30 (3)	N4—C32—H32B	109.5
Cu3—Cu4—I3	60.95 (2)	H32A—C32—H32B	109.5
Cu2—Cu4—I3	59.205 (17)	N4—C32—H32C	109.5
I2—Cu4—I3	117.93 (2)	H32A—C32—H32C	109.5
Cu1—Cu4—I3	109.405 (17)	H32B—C32—H32C	109.5
Cu4—I1—Cu2	60.49 (2)	N4—C33—C34	116.2 (3)
Cu4—I1—Cu1	61.070 (17)	N4—C33—H33A	108.2
Cu2—I1—Cu1	58.366 (16)	C34—C33—H33A	108.2
Cu4—I2—Cu3	58.773 (17)	N4—C33—H33B	108.2
Cu4—I2—Cu1	60.43 (2)	C34—C33—H33B	108.2
Cu3—I2—Cu1	63.26 (2)	H33A—C33—H33B	107.4
Cu2—I3—Cu4	59.81 (2)	C35—C34—C39	117.8 (3)
Cu2—I3—Cu3	62.04 (2)	C35—C34—C33	120.7 (3)
Cu4—I3—Cu3	58.208 (14)	C39—C34—C33	121.5 (3)
Cu3—I4—Cu1	64.025 (19)	C36—C35—C34	121.6 (3)
Cu3—I4—Cu2	62.53 (2)	C36—C35—H35A	119.2
Cu1—I4—Cu2	58.665 (15)	C34—C35—H35A	119.2
N1—C1—H1A	109.5	C37—C36—C35	119.8 (3)
N1—C1—H1B	109.5	C37—C36—H36A	120.1
H1A—C1—H1B	109.5	C35—C36—H36A	120.1
N1—C1—H1C	109.5	C38—C37—C36	119.8 (3)
H1A—C1—H1C	109.5	C38—C37—H37A	120.1
H1B—C1—H1C	109.5	C36—C37—H37A	120.1
N1—C2—H2A	109.5	C37—C38—C39	120.2 (3)
N1—C2—H2B	109.5	C37—C38—H38A	119.9
H2A—C2—H2B	109.5	C39—C38—H38A	119.9
N1—C2—H2C	109.5	C38—C39—C34	120.8 (3)
H2A—C2—H2C	109.5	C38—C39—H39A	119.6
H2B—C2—H2C	109.5	C34—C39—H39A	119.6
N1—C3—C4	115.5 (2)	C2—N1—C1	108.5 (3)
N1—C3—H3A	108.4	C2—N1—C3	110.9 (3)
C4—C3—H3A	108.4	C1—N1—C3	110.7 (3)
N1—C3—H3B	108.4	C2—N1—Cu1	108.97 (19)
C4—C3—H3B	108.4	C1—N1—Cu1	108.2 (2)
H3A—C3—H3B	107.5	C3—N1—Cu1	109.50 (18)
C5—C4—C9	118.2 (3)	C12—N2—C11	108.6 (3)
C5—C4—C3	121.5 (3)	C12—N2—C13	111.0 (3)
C9—C4—C3	120.4 (3)	C11—N2—C13	111.3 (3)
C4—C5—C6	120.6 (3)	C12—N2—Cu2	109.2 (2)
C4—C5—H5A	119.7	C11—N2—Cu2	108.5 (2)
C6—C5—H5A	119.7	C13—N2—Cu2	108.2 (2)
C7—C6—C5	120.5 (3)	C21—N3—C22	107.7 (3)
C7—C6—H6A	119.7	C21—N3—C23	110.7 (3)
C5—C6—H6A	119.7	C22—N3—C23	110.4 (3)
C6—C7—C8	119.6 (3)	C21—N3—Cu3	112.0 (2)
C6—C7—H7A	120.2	C22—N3—Cu3	107.9 (2)
C8—C7—H7A	120.2	C23—N3—Cu3	108.1 (2)
C7—C8—C9	119.9 (3)	C32—N4—C31	109.2 (3)
C7—C8—H8A	120.1	C32—N4—C33	111.3 (3)
C9—C8—H8A	120.1	C31—N4—C33	111.4 (3)
C8—C9—C4	121.1 (3)	C32—N4—Cu4	109.7 (2)
C8—C9—H9A	119.4	C31—N4—Cu4	107.7 (2)
C4—C9—H9A	119.4	C33—N4—Cu4	107.49 (19)
